# Irritable Bowel Syndrome: The role of the Psychiatry

**DOI:** 10.1192/j.eurpsy.2022.1220

**Published:** 2022-09-01

**Authors:** A. Fraga, B. Mesquita, D. Esteves-Sousa, M. Albuquerque, J. Facucho-Oliveira, P. Espada-Santos, P. Cintra, A. Moutinho

**Affiliations:** Hospital de Cascais, Psychiatry, Alcabideche, Portugal

**Keywords:** psychiatry, Treatment, Irritable Bowel Syndrome, Functional

## Abstract

**Introduction:**

Irritable bowel syndrome (IBS) is the most common functional gastrointestinal disorder, affecting about 20% of people worldwide. This complex and multifaceted disorder has been proposed as a system disease involving not only individual systems including the nervous, endocrine, imune, digestive, microbiota and the environment but also the interactions of these systems. The aetiology of IBS is complex and incompletely understood and this disease are frequently associated with a comorbid psychiatric disease. Current treatment is symptom-directed, rather than based on underlying pathophysiological mechanisms.

**Objectives:**

The authors elaborate a narrative literature review to identify the pathophysiology and therapeutic approach of IBS.

**Methods:**

Pubmed databased searched using the therms “psychiatry”, “irritable bowel syndrome” and “treatment”.

**Results:**

The IBS is the most common and best described of the functional bowel disorders, which represents a considerable therapeutic challenge. Studies looked at the efficacy of fibre, antispasmodics and peppermint oil in the treatment of IBS found moderately effectiveness in the treatment of global symptoms. Elimination diets are helpful in improving IBS. There is evidence that a low-FODMAP diet can have a favorable impact on IBS symptoms, especially abdominal pain, bloating and diarrhea with improved irritable bowel syndrome symptoms and quality of life. Among the currently available classes of drugs for the treatment of IBS, antidepressants such as selective serotonin releasing inhibitors and tricyclic antidepressants are useful because of their analgesic properties, independent of their mood-improving effects.

**Conclusions:**

Evidence suggest that antidepressants might be useful for treatment symptom of IBS however further investigation is required.

**Disclosure:**

No significant relationships.

